# Potential role of orexin A binding the receptor 1 for orexins in normal and cryptorchid dogs

**DOI:** 10.1186/s12917-018-1375-6

**Published:** 2018-02-27

**Authors:** Giovanna Liguori, Caterina Squillacioti, Loredana Assisi, Alessandra Pelagalli, Alfredo Vittoria, Anna Costagliola, Nicola Mirabella

**Affiliations:** 1 Department of Veterinary Medicine and Animal Production, University of Naples “Federico II”, Via Delpino 1, 80137 Naples, Italy; 2 Department of Biology, University of Naples “Federico II”, Via Mezzocannone 6, 80134 Naples, Italy; 3 Department of Advanced Biomedical Sciences, University of Naples “Federico II”, Via Pansini 5, 80131 Naples, Italy; 4 Institute of Biostructures and Bioimages, National Research Council, Via De Amicis 95, 80131 Naples, Italy

**Keywords:** Orexin A, Receptor 1 for orexins, Dog testis, Cryptorchidism, Steroidogenesis

## Abstract

**Background:**

Cryptorchidism is one of the most common birth disorders of the male reproductive system identified in dogs and other mammals. This condition is characterised by the absence of one (unilateral) or both (bilateral) gonads from the scrotum. The peptides orexin A (OxA) and B (OxB) were obtained by post-transcriptional proteolytic cleavage of a precursor molecule, called prepro-orexin. These substances bind two types of G-coupled receptors called receptor 1 (OX1R) and 2 (OX2R) for orexins. OX1R is specific to OxA while OX2R binds the two peptides with equal affinity. Orexins modulate a great variety of body functions, such as the reproductive mechanism. The purpose of the present research was to study the presence of OxA and its receptor 1 and their possible involvement in the canine testis under healthy and pathological conditions.

**Methods:**

This study was performed using adult male normal dogs and male dogs affected by unilateral cryptorchidism. Tissue samples were collected from testes and were divided into three groups: normal, contralateral and cryptic. The samples were used for immunohistochemistry, Western blot and in vitro tests for testosterone evaluation in normal and pathological conditions.

**Results:**

OxA-immunoreactivity (IR) was described in interstitial Leydig cells of the normal gonad, and Leydig, Sertoli cells and gonocytes in the cryptic gonad. In the normal testis, OX1R-IR was described in Leydig cells, in pachytene and second spermatocytes and in immature and mature spermatids throughout the stages of the germ developing cycle of the male gonad. In the cryptic testis OX1R-IR was distributed in Leydig and Sertoli cells. The presence of prepro-orexin and OX1R was demonstrated by Western blot analysis. The incubation of fresh testis slices with OxA caused the stimulation of testosterone synthesis in the normal and cryptic gonad while the steroidogenic OxA-induced effect was cancelled by adding the selective OX1R antagonist SB-408124.

**Conclusions:**

These results led us to hypothesise that OxA binding OX1R might be involved in the modulation of spermatogenesis and steroidogenesis in canine testis in healthy and pathological conditions.

## Background

Cryptorchidism is considered a reproductive disease characterised by the failure of one or both testes to descend towards the scrotum [1, 2] and is defined a testicular dysgenesis syndrome together with hypospadias, germ cell tumour, and subfertility [3–5]. It is commonly classified as a birth defect of the male genital tract in mammals, particularly studied in dogs and humans. In dogs, unilateral cryptorchidism, particularly involving the right gonad [1] is more frequently detected [1, 6, 7]. In unilateral cryptorchidism, the abdominal testis may be involved in the risk of neoplasms such as Sertoli cell tumours and seminomas [8, 9]. The abdominal localisation of the retained gonad is histologically characterised by disruption of the germinal epithelium [10].

Orexins A (OxA) and B (OxB) are two peptides of hypothalamic origin [11, 12]. These peptides derive from a post-transcriptional proteolytic cutting of a common precursor called prepro-orexin. Orexins are involved in regulating many biological functions by binding two types of G-coupled transmembrane receptors called receptor 1 (OX1R) and 2 (OX2R) for orexins [11–17].

More recently, scientific thinking has focused on the role of orexins in the mammalian genital tract under normal and pathological conditions. In particular, the presence of OxA, prepro-orexin and OX1R has been described in the rat [18] and alpaca epididymis [19] and in the testis of the rat, alpaca and mouse [20–24], in the urethro-prostatic complex of cattle [25], and in the normal, hyperplastic and neoplastic prostate of human beings [26–28]. The expression of OX1R mRNAs has been detected in the testis of sheep [29], chicken [30], in primary rat Leydig cells [31] and in the human male genital tract [32]. The detection of mRNA codifying for prepro-orexin was found in rat testis [33–35] and human epididymis and penis [33]. The peptides OxB and OX2R were detected in the testis of rat [36] and alpaca (*Vicugna pacos*) [37] and the OX2R mRNAs were demonstrated in the human male genital tract [32] and prostate [38].

To date, there has been no evidence of the orexin complex and its potential role in the testis of normal and cryptorchid dogs. The expression of OxA and OX1R in normal and pathological conditions of the male gonad and their potential role in the reproductive system are still debated. The aim of our research was to characterise OxA and OX1R expressions in normal and cryptic testis of the dog in order to investigate their involvement in clinico-pathological conditions and their possible steroidogenic effect. Such characterisation could make a new contribution regarding the complex biochemical (autocrine/paracrine) mechanisms operating at the level of the male gonad.

## Methods

### Antibodies and chemicals

Mouse anti-OxA (MAB763) monoclonal antibody and its synthetic peptides were obtained from R&D Systems (Abingdon, UK) and from Tocris Bioscience (Bristol, UK), respectively; rabbit polyclonal anti-OX1R antibody (PAB8017) from Abnova (Taipan, Taiwan) and the synthetic blocking peptide (ab188501) from Abcam (Cambridge, UK); rabbit polyclonal anti-prepro-orexin antibody (AB3096), its blocking peptide (AG774) from Millipore (Temecula, CA, USA); monoclonal anti b-actin antibody (JLA20 CP01) from Calbiochem, San Diego, CA, USA; biotinylated goat anti-mouse (BA-9200) and goat anti-rabbit (BA-1000) secondary antibodies and avidin–biotin complex (PK-6105) from Vector Laboratories (Burlingame, CA, USA); horseradish peroxidase goat anti-rabbit IgG (A-0545) from the Sigma Chemical Co. (St. Louis, MO, USA); and marker proteins from Prosieve, Lonza, Rockland, ME, USA. The peptide OxA (003–30) was purchased from Phoenix Pharmaceuticals and the selective non-peptide orexin OX1R antagonist SB-408124 (1963) from Tocris Bioscience (Bristol, UK); the luteinizing hormone (LH) from sheep pituitary (L5269); EIA kit for testosterone determination from Adaltis (Bologna, Italy). The Super Signal West Pico Chemiluminescent Substrate was obtained from Thermo Scientific (Pierce, Rockford, IL, USA), and Kodak Gel Logic 1500 imaging system from Celbio (Milan, Italy).

### Animals and tissue collection

The animals employed in this research were five adult normal male dogs and five cryptorchid dogs (unilateral cryptorchidism; in three subjects the testis was retained in the abdomen and in two in the inguinal canal) aged between 2 and 8 years. All owners gave verbal consent to perform surgical procedures, collection of the samples and animals were not involved in any clinical trials or treatments. Animal care was guaranteed during the surgical procedures and the experimental research was approved by the Ethical Animal Care and Use Committee of the University of Naples Federico II, Department of Veterinary Medicine and Animal Production, Naples, Italy (no. 0005275). The gonads were rapidly collected and tissue specimens were divided into three groups: normal testis (testis from normal dogs, N), contralateral testis (scrotal testis from cryptorchid subjects, CL) and cryptic testis (retained gonad from cryptorchid subjects, CR). Part of the tissue specimens was cut into small samples which were immersed in Bouin’s fluid for paraplast embedding as described in detail elsewhere [39] and another part of the tissue specimens was frozen in liquid nitrogen and stored at − 80 °C until used for Western blotting analysis and in vitro tests for testosterone evaluation.

### Immunohistochemistry

After Paraplast embedding the tissue blocks were cut into 6 μm thick sections. After rehydration, the antigen retrieval method was applied to the sections as previously described by Liguori et al. (2017) [36]. The immunohistochemical method performed for the present research was the avidin–biotin–peroxidase complex (ABC) and the protocol used was that previously described by Liguori et al. (2013) and De Luca et al. (2014) [40, 41]. The sections were covered with mouse monoclonal anti-OxA and rabbit polyclonal anti-OX1R primary antibodies, diluted 1:200, applied on sections overnight at 4 °C. The day after, after three PBS washes the sections were covered with biotinylated goat anti-mouse IgG or goat anti-rabbit IgG, both 1:200 diluted, for 30 min at room temperature. Incubation was then performed in ABC reagent for 30 min. The staining was completed with 3–3′ diaminobenzidine. Sections were counterstained with haematoxylin in order to better localise the immunoreactive materials. The specificity of the experiment was evaluated by omitting and preabsorbing the two antibodies with an excess (100 μg/ml) of the relative antigens (data not shown). No immunoreactivity was found. The sections were observed by a Nikon Eclipse E 600 light microscope, and pictures were taken by a Nikon Coolpix 8400 digital camera.

### Western blotting analysis

Tissue samples were first broken down mechanically using an Ultra-Turrax homogenizer in RIPA lysis buffer (0.1 mM PBS, 1% Nodinet P-40, 0.1% Sodium Dodecyl Sulphate 0.05% (SDS) deoxycholate, 1 lg/ml leupeptin and 1 lg/ml phenylmethylsulphonyl fluoride (PMSF) and then centrifuged at 16000 x g for 20 min at 4 °C. The proteins of the supernatant were separated using SDS/polyacrylamide gel electrophoresis (SDS/PAGE) (15% polyacrylamide) under reducing conditions and then transferred onto immunoblot nitrocellulose membrane as described elsewhere [42, 43]. Blocking of non-specific binding is achieved by placing the membrane in a buffer (5% BSA and 0.3% Tween 20 in PBS) and then a diluted solution of specific primary antibodies (anti-prepro-orexin and anti-OX1R; diluted 1:500) is incubated with the membrane for 2 h at room temperature under gentle agitation. After rinsing the membrane to remove unbound primary antibodies, the membranes were exposed to peroxidase-conjugated goat anti-rabbit IgG (diluted 1:2000 in blocking solution) for 1 h at room temperature. Molecular weight approximations are taken by comparing the stained bands to that of the Marker proteins (coloured protein molecular weight markers). The protein bands were detected by chemiluminescence, and the image was captured by Kodak Gel Logic 1500 digital imaging equipment. The specificity of the experiment was evaluated by preabsorbing the antibodies with an excess (100 μg/ml) of the relative antigen. For a loading control, stripping and re-probing of the blots with an anti b-actin monoclonal antibody were performed.

### Testosterone evaluation

#### Tissue incubations

The role played by OxA in regulating steroidogenesis was evaluated by incubating testicular slices as reported in a previous paper [21], with minor modifications. The removed testes of each group were decapsulated and cut into pieces, each piece weighing 250 ± 7 mg. Testicular slices (2 slices/well) were incubated with 2 ml Krebs-Ringer bicarbonate (KRB) buffer (pH 7.4) containing 10 mM glucose, 100 μM bacitracin, 0.1% ascorbic acid, and 0.1% bovine serum albumin. The fresh slices were subjected to constant shaking (60 cycles per min) for 60 min in an atmosphere of 95% 0_2_/5% CO_2_ at 37 °C. Then the samples were treated with 1 ml of fresh KRB buffer containing 1 nM of OxA alone and/or OxA with its antagonist for 24 h. Controls were obtained by adding to the slices the buffer alone or 1 nM luteinizing hormone (LH). Testosterone levels were evaluated as reported below.

#### Determination of testosterone levels

After separation of the medium, it was subjected to testosterone determination using an enzyme immunoassay kit (EIA). The following coefficients of variability of testosterone determination were identified: sensitivity 6 pg, intra-assay variability 5.3% and inter-assay variability 7.5%. The process of extraction was first made by mixing vigorously the medium with ethyl ether (1:10, *v*/v). After centrifugation at 3000 g for 10 min, the ether fraction was obtained and separated. The pooled ether fractions were then evaporated to dryness and the residue washed with a 0.5 ml sodium phosphate buffer 0.05 M (pH 7.5), containing 10 mg/ml BSA. Finally, testosterone immunoassay was performed, as described previously [22]. The rate of testosterone recovery obtained from testis was about 80%. The levels of testosterone were expressed as normalised values per g incubated tissue. The in vitro tests were performed in triplicate and data were expressed as means ± SD.

### Statistical analysis

The results obtained were compared by analysis of variance by using the multiple comparison Duncan’s test and group comparison Student’s t-test. Descriptive data were expressed as mean and standard deviation (SD). The data obtained were considered statistically significant at *p* < 0.01 and *p* < 0.05.

## Results

### Immunohistochemical evaluation of OxA and OX1R in normal and cryptorchid testes of dogs

**Fig. 1:**
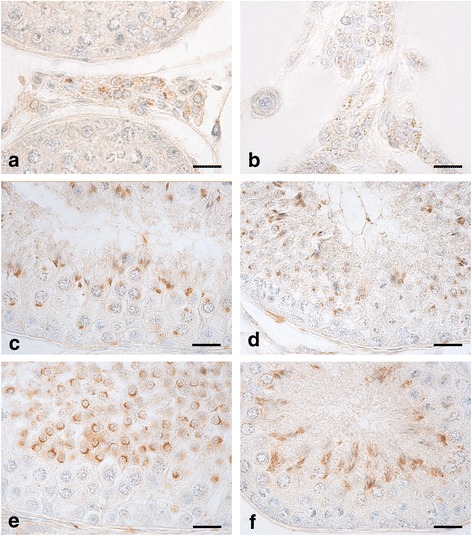
OxA- and OX1R-IR in cytotypes of N testes of dogs. **a**, **b**: a cluster of Leydig cells containing different quantities of positive granules for both the peptides scattered in their cytoplasm in the N group of canine testis. **c**, **d**: OX1R-IR, as a single, intensely stained, granular structure roundish in shape, is contained in the perinuclear cytoplasm of some pachytene (**c**) and secondary (**d**) spermatocytes. **e**, **f**: round (**e**) and elongated (**f**) spermatids intensely stained by the anti-OX1R antibody. Avidin–biotin immunohistochemical technique. Bars: 20 μm

The immunohistochemical results are summarised in Table 1. OxA (Fig. 1a)- and OX1R (Fig. 1b)- immunoreactivity (IR) was found in Leydig cells in the N group of canine testes. The reactive material showed a granular aspect and cytoplasmic localisation in both cases. Leydig cells (Fig. 1a) were numerous and often organised in small groups composed by differently stained and negative cells. Additionally, OX1R stained positive in the following cells in N group of the canine testes: pachytene (Fig. 1c) and secondary (Fig. 1d) spermatocytes as well as young (round) (Fig. 1e) and mature (elongated) (Fig. 1f) spermatids. In pachytene (Fig. 1c) and secondary (Fig. 1d) spermatocytes, positive immunolabelling for OX1R was granular and perinuclear. During spermatid maturation the positive material changed its appearance following the morphological transformation of the cell which is known to be round in the young elements, becoming progressively elongated in the older ones. In particular, in round spermatids (Fig. 1e), the positive immunolabelling for OX1R seemed to be localised in the acrosome body with a semilunar and/or perinuclear aspect. In elongated spermatids (Fig. 1f) the positive signal was granular and cytoplasmic. OxA- and OX1R-IR in the CL group was similar to that described above for that of N (data not shown).

**Table 1:** Distribution of OxA- and OX1R-immunolabellihng in the normal and cryptorchid dog

	Leydig cells	Sertoli cells	Early germ cells	Pachytene spermatocytes	Secondary spermatocytes	Round spermatids	Elongated spermatids
1a. OxA-immunolabelling							
N group	+	–	–	–	–	–	–
CL group	+	–	–	–	–	–	–
CR group	+	+	+	–	–	–	–
1b. OX1R-immunolabelling							
N group	+	–	–	+	+	+	+
CL group	+	–	–	+	+	+	+
CR group	+	+	–	–	–	–	–

+: presence of immunolabelling, −: absence of immunolabelling

**Fig. 2:**
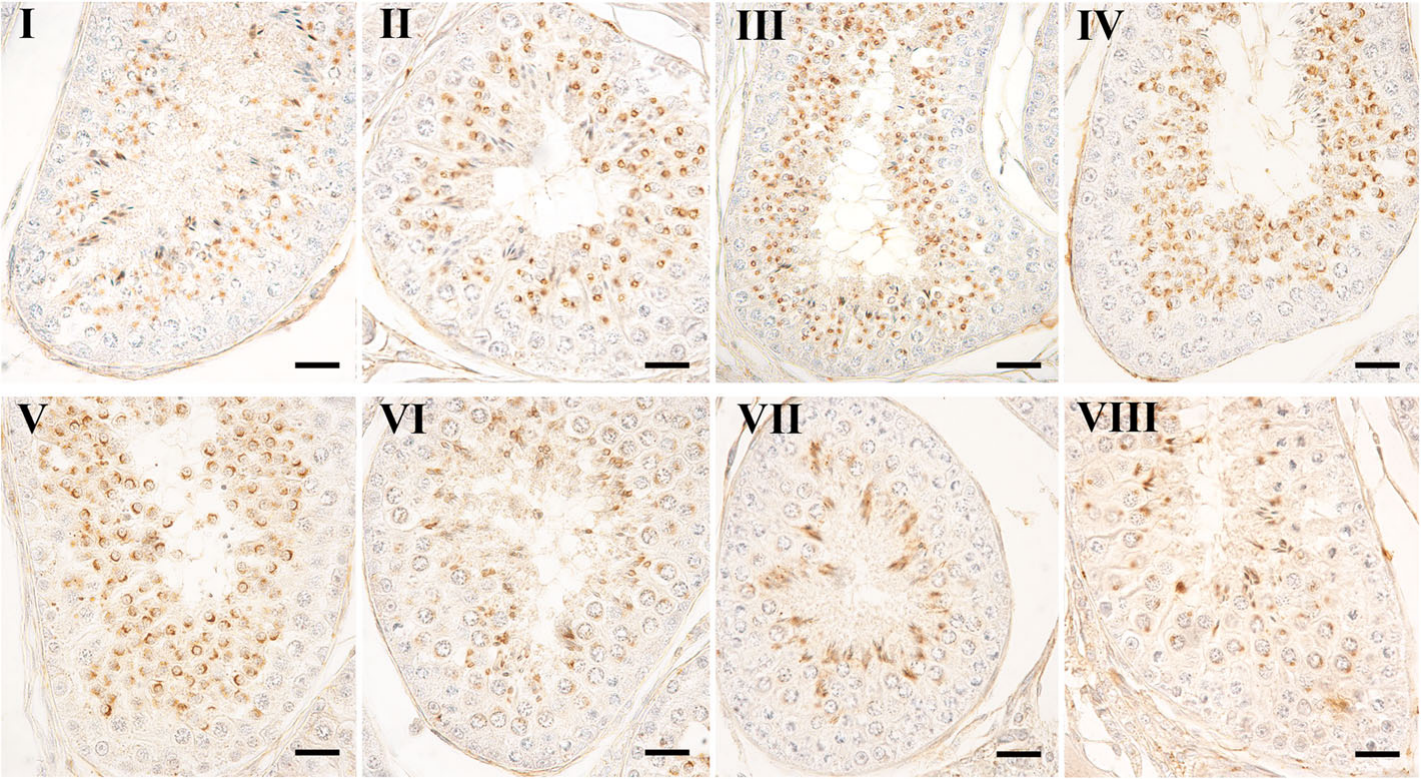
OX1R containing cytotypes along the seminiferous epithelium cycle of dog testis. An accurate examination of haematoxylin-counterstained sections revealed that positive cytotypes were present in all stages of the seminiferous epithelium cycle ranging from Stage I to VIII. Avidin–biotin immunohistochemical technique. Bars: 20 μm

OX1R-IR-containing cell types were found at all stages of the seminiferous epithelium cycle of the N group (Fig. 2) according to the cycle described by Soares and co-workers (2009) [44]. Positive round spermatids were intensely stained from stages I to V of the cycle; immunoreactive pachytene spermatocytes and developing spermatids were typical of the stage VI of the cycle; elongated spermatids were described in stages VII and VIII of the cycle; and especially in stage VIII positive secondary spermatocytes were described.

**Fig. 3:**
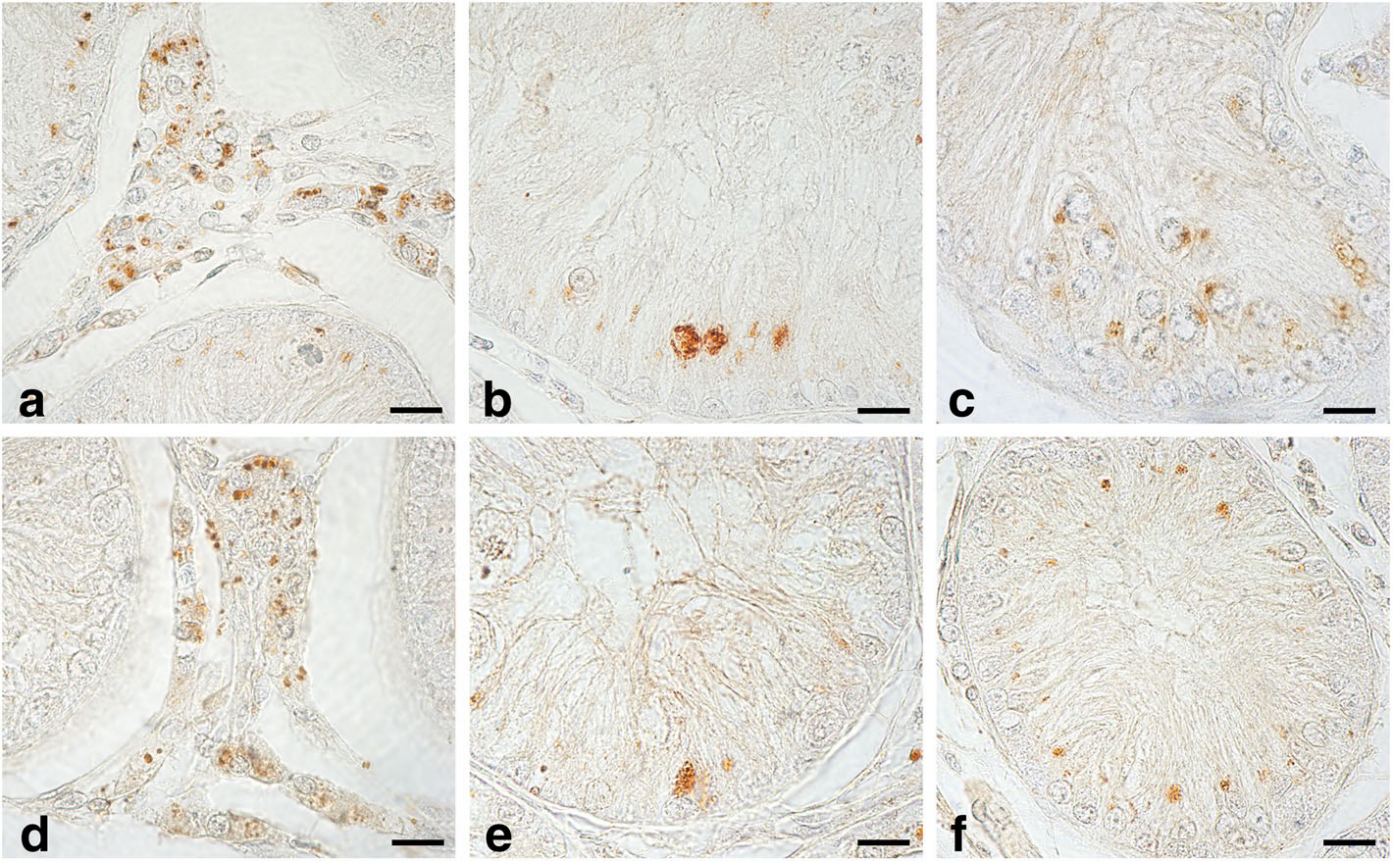
OxA- and OX1R-IR in cytotypes of the CR group of dog testis. **a**, **d**: Leydig cells showing OX1R and OxA containing condensed granular material were intensely stained. **b**, **e**, **f**: intensely stained secretory granules condensed in the perinuclear portion of the cytoplasm of Sertoli cells. **c**: rare early germ cells immunoreactive to weakly stained OxA in the tubular compartment of the CR group. Avidin–biotin immunohistochemical technique. Bars: 20 μm

OxA (Fig. 3a-c)- and OX1R (Fig. 3d-f)-IR were also seen in the CR group of canine testes. The expression of both peptides was seen in: Leydig cells (Fig. 3a,d), Sertoli cells (Fig. 3b, e, f) and in early germ cells (Fig. 3c), the latter being positive only to OxA. Groups of Leydig cells were intensely stained (Fig. 3a, d). In particular, seminiferous tubules of the CR group were composed mostly by Sertoli cells and early germ cells. In Sertoli cells a perinuclear expression of OxA was observed (Fig. 3b, e, f). In early germ cells (Fig. 3c) OxA-IR assumed the appearance of roundish granules which were localised in a perinuclear position.

### Expression of prepro-orexin and OX1R in tissue extracts

**Fig. 4:**
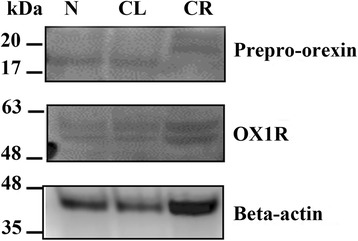
Expression of prepro-orexin and OX1R in tissue homogenates. Prepro-orexin (16 kDa) was detected in N, CL and CR testes. OX1R (50 kDa) was detected in N, CL and CR testes. Beta-actin was used as a loading control. Molecular weight standards are expressed in KDa

The results of Western blot analysis are shown in Fig. 4. Tissue extracts of the N, CL and CR testes of the dog reacted with the anti-prepro-rexin and anti–OX1R antibodies. Testicular extracts reacted with the anti-prepro-rexin and recognized a major protein band of approximately 16 kDa from tissue homogenates (Fig. 4). Furthermore, the anti-OX1R antibody recognized a major protein band measuring approximately 50 kDa (Fig. 4). These findings suggest that canine testes express prepro-orexin and OX1R supporting the immunohistochemical results.

### Testosterone evaluation in OxA treated testis slices

**Fig. 5:**
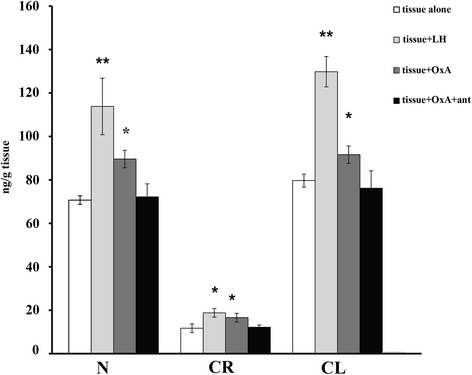
Testosterone evaluation by incubation of dog testis slices with LH, OxA and OxA with antagonist. Tissues from N, CR and CL groups were incubated and testosterone level in the media was monitored after 24 h. Tissue alone was used as control of the experiment. Values are normalised per gram of incubated tissue. Data expressed as mean ± SEM,*P < 0.05, **P < 0.01 vs. corresponding controls (ANOVA followed by Student’s t test)

Assessment of LH, OxA alone and combined with its antagonist effects on steroidogenesis in vitro was carried out in testis of adult male dogs. Fig. 5 shows the results from an in vitro experiment in which 1 nM concentration of LH, or OxA alone and/or of OxA with its antagonist was added to a medium containing each group of dog testicular slices. It is evident that LH stimulated significantly the basal steroidogenesis in the normal gonad. Specifically, after 24 h testosterone production was higher than that of the control (from 70 ± 2 to 113.8 ± 3 ng/g tissue, *p* < 0.01 versus control). Similarly, after 24 h incubation with OxA, the testosterone increase was significantly higher than that of the control (from 70 ± 2 to 89 ± 4 ng/g tissue, *p* < 0.01 versus control). On the contrary, incubations with 1 nM concentration of OxA with its antagonist caused a decrease in testosterone level (from 89 ± 4 to 72 ± 6 ng/g tissue, *p* < 0.05 versus OxA). The subsequent results showed an in vitro experiment in which 1 nM concentration of LH, or OxA alone and/or OxA with its antagonist was added to a medium containing the CR group of canine testicular slices. The first evidence was that the testosterone levels were lower in all medium in accordance with the tissue degeneration observed in histology tests as well. However, LH proved able to stimulate basal testosterone secretion significantly. Specifically, after 24 h testosterone synthesis was higher than that of the control (from 11.7 ± 2 to 18.8 ± 3 ng/g tissue, *p* < 0.05 versus control). After 24 h of incubation with OxA, there was also a significant testosterone increase over that of the control (from 11.7 ± 2 to 16.6 ± 1 ng/g tissue, *p* < 0.05 versus control). This increase was annulled by the presence of the antagonist (from 16.6 ± 1 to 12.2 ± 2 ng/g tissue).

The results of the in vitro experiment in which 1 nM concentration of LH, or of OxA alone and/or OxA with its antagonist was added to a medium containing the CL group of canine testicular slices were very similar to those shown in normal tissue. LH proved able to stimulate basal testosterone secretion significantly. Nevertheless, after 24 h testosterone synthesis was higher than that of the control (from 79.7 ± 3 to 129.8 ± 7 ng/g tissue, *p* < 0.01 versus control). Incubation with OxA also caused a significant testosterone increase compared to that of the control (from 79.7 ± 3 to 91.6 ± 4 ng/g tissue, *p* < 0.05 versus control). The presence of the antagonist annulled this increase (from 91.6 ± 4 to 72.2 ± 8 ng/g tissue *p* < 0.05 versus OxA).

## Discussion

In this research, the localisation of OxA and OX1R was found in many testicular cytotypes of the N, CL and CR testes of dogs by means of immunohistochemistry. OxA- and OX1R-IR were described in the interstitial Leydig cells and in the tubular compartment of all examined groups. Western blotting analysis was performed to demonstrate the presence of both the peptides in all tissue homogenates.

The effect of OxA on Leydig cell steroidogenesis was previously described in rat, alpaca and mouse testis by different research groups [21, 22, 24, 31, 34, 35]. OxA evoked the increase of testosterone synthesis, antagonising the steroidolitic effect of Müllerian Inhibiting Substance (MIS), which was secreted from Sertoli cells. The two substances counteracted their reciprocal effects. This mechanism represented the main molecular event of OxA-steroidogenic action in mammalian testis. In vitro experiments were carried out in this research, incubating slices from N, CL and CR testes with OxA which enhanced testosterone synthesis in all testicular groups. OX1R seems to have a pivotal role in this mechanism: the incubation of testicular slices with its antagonist SB-408124 made the OxA-induced steroidogenic effect disappear. These findings definitively confirmed the effect of OxA in stimulating testosterone synthesis. This steroidogenic action in the CR group is less than that described for N and CL testes. Testosterone synthesis in Leydig cells is mainly regulated by the pituitary hormone LH, which acts on target cells by binding its receptors (LHR) [45]. In cryptorchidism LH-induced testosterone production is lower in the cells of retained gonads than scrotal ones [46–48]. The decreased testosterone concentration in the undescended testes has been ascribed to reduced LHR expression in rats [49, 50], rams [51] and men [52]. Another substance involved in regulating steroidogenesis is aromatase, which promotes the conversion of androgens to oestrogens [53]. In cryptorchid stallions, mice and dogs an increased aromatase immunoexpression and higher oestradiol levels were demonstrated [54–56]. Therefore, the decrease in testosterone levels might be caused not only by a reduction in LHR expression but also by the increased conversion of androgens into oestrogens. Several studies were previously performed regarding the relationship between orexins/LH and orexin/aromatase while no data are available on OxA and LHR. Orexins show a stimulatory effect on the secretory activity of the GnRH/LH axis in rats and men [57, 58] and decrease the amplitudes of LH pulses in castrated camels deprived of the negative feedback of testosterone [59]. Moreover, the central injection of orexin significantly decreased aromatase mRNA levels in the hypothalamus of androgenised female rats [60] and increased aromatase mRNA levels and oestradiol concentrations in the hypothalamus of wild-type male rats [61]. Taking these findings together, we could hypothesise that the stimulation of OxA-evoked testosterone secretion in cryptorchid testis might be due to: 1. upregulation of LHR expression on OxA-induced Leydig cells and/or 2. downregulation of aromatase expression, which converts testosterone and androstenedione into oestrogens. That said, these are only hypotheses that need to be further investigated. In CL testes the low increase in steroidogenesis production compared to those of N might be due to a vicarious action of the CL gonad to make up for the reduced testosterone production of that of CR.

In the tubular compartment OxA- and OX1R- IR were previously described in numerous cytotypes of rat [20, 21, 35], alpaca [22] and mouse [24] testes. In the canine testes of groups N and CL, OxA-IR was only detected in the Leydig cells, while OX1R-IR in pachytene and secondary spermatocytes and spermatids from immature to mature forms. The positive cytotypes were described in the whole seminiferous epithelium cycle of the dog testis (from stages I to VIII). These data suggest that the seminiferous tubules of mammalian testis are characterised by different cytotypes able to produce and/or internalise OxA. Moreover, OxA might be involved in spermatogenesis regulation, via OX1R, in the normal male gonads.

Our research also detected OxA and OX1R containing cytotypes in the tubular compartment of the CR group, in early germ cells and Sertoli cells. Early germ cells may be arrested gonocytes. Cryptorchidism has detrimental effects on spermatogenesis [62] and usually might cause clinical infertility associated with a serious decrease in spermatozoa production. Germ cell apoptosis seemed to play a pivotal role in regulating N and CR testes [63–65]. Previous studies have demonstrated that cryptorchidism induced elevation of testicular temperature which in turn affected the morphology and function of Sertoli cells with a high grade of hyperplasia of this cytotype [66] and in the testis of monkey and rat regain undifferentiated features like immature state via activation of the extracellular signal-regulated kinases 1/2 (ERK 1/2) mitogen-activated protein kinases (MAPK) pathway [67]. This condition allows some early germ cells to persist which might be histologically similar to neonatal testis. Joshi and Singh (2016) [23] described the presence of OxA and OX1R in neonatal testis of mice and showed that the expression of OxA and OX1R decreased after OX1R antagonist treatment. The inhibition of this binding may interfere with the downstream signalling pathway leading to the down-regulation of the stem cell factor (SCF). The SCF/c-kit system has been proved to stimulate DNA synthesis and cell growth, acting as anti-apoptotic factor on primordial germ cells and spermatogonia and preparing germ cells to enter meiosis [68]. In analogy with the findings described above in neonatal testis by Joshi and Singh (2016) [23], OxA, via OX1R, may not inhibit SCF secretion by the undifferentiated Sertoli cells of the cryptic gonad, with a consequent anti-apoptotic effect on early germ cells. The failure to eliminate abnormal early germ cells by using an anti-apoptotic mechanism could lead to retention of defective cells involved in the formation of human testicular germ cell tumours such as seminoma [69].

The role of orexins as a pro-apoptotic or anti-apoptotic factor is still debated. In particular, orexins have a pro-apoptotic role in colon cancer cell lines including HT-29 [70, 71], the human neuroblastoma SK-N-MC cells [70], rat C6 glioma cells [72], the rat pancreatic cancer cell line AR42J [73] and in prostate cancer [26]. The signal transduction orexins-induced pathway may be involved in regulating cell survival. OxA promoted proliferation and viability in human gastric cancer cells SGC-7901 with activation of the ERK1/2-MAPK pathway [74], human adrenocortical adenomas [75], in immortalised primary embryonic rat hypothalamic R7 cells [76], 3 T3-L1 preadipocytes [77] and in rat hepatocytes [78]. The peptide OxA and its receptor 1 appear to share some similarities in N and CR testis of the dog with other peptides such as urocortins, which might play a role in spermatogenesis and steroidogenesis regulation also in the male gonad of the dog [79]. The blockage of OxA binding OX1R by its own antagonist might be a new therapeutic target involved in inhibiting the abnormal germ cell growth OxA-induced in cryptorchidism.

## Conclusions

In conclusion, these results led us to demonstrate that OxA and its receptor 1 are expressed in the normal and cryptic testis of the dog. In particular, in the interstitial compartment a steroidogenic OxA effect, via OX1R, by means of an autocrine/paracrine mechanism may be hypothesised. Moreover, in the tubular compartment the peptide is involved in spermatogenesis regulation in normal testis and a proliferative action in the cryptic gonad. The latter mechanism might acquire great importance in the neoplastic transformation of the retained male gonad. Although the molecular mechanisms of the OX1R-mediated anti-apoptotic effect of OxA remain to be elucidated, these may have important implications in targeting new therapies for reproductive diseases in animals and humans.

## Abbreviations

CL: Contralateral testis
CR: Cryptic testis
EIA: Enzyme immunoassay
ERK 1/2: Extracellular Signal-Regulated Kinases 1/2
hCG: human corionic gonadotropin
IR: Immunoreactivity
LH: Luteinizing hormone
LHR: Luteinizing hormone receptor
MAPK: Mitogen-Activated Protein Kinases
MIS: Müllerian Inhibiting Substance
N: Normal testis
OX1R: receptor 1 for orexins
OX2R: receptor 2 for orexins
OxA: Orexin A
OxA: Orexin B
SCF: Stem cell factor
